# Effect of Active Site Modification towards Performance Enhancement in Biopolymer κ-Carrageenan Derivatives

**DOI:** 10.3390/polym12092040

**Published:** 2020-09-08

**Authors:** Mohd Hafiz Abu Bakar, Nur Hidayah Azeman, Nadhratun Naiim Mobarak, Mohd Hadri Hafiz Mokhtar, Ahmad Ashrif A Bakar

**Affiliations:** 1 Photonics Technology Laboratory, Department of Electrical, Electronic & Systems Engineering, Faculty of Engineering and Built Environment, Universiti Kebangsaan Malaysia, Bangi 43600, Selangor, Malaysia; hafiz.bujei95@gmail.com (M.H.A.B.); hadri@ukm.edu.my (M.H.H.M.); 2 Department of Chemical Sciences, Faculty of Science and Technology, Universiti Kebangsaan Malaysia, Bangi 43600, Selangor, Malaysia; nadhratunnaiim@ukm.edu.my

**Keywords:** polysaccharide, biopolymer, carrageenan, functionalization, active site

## Abstract

This research demonstrates a one-step modification process of biopolymer carrageenan active sites through functional group substitution in κ-carrageenan structures. The modification process improves the electronegative properties of κ-carrageenan derivatives, leading to enhancement of the material’s performance. Synthesized succinyl κ-carrageenan with a high degree of substitution provides more active sites for interaction with analytes. The FTIR analysis of succinyl κ-carrageenan showed the presence of new peaks at 1068 cm^−1^, 1218 cm^−1^, and 1626 cm^−1^ that corresponded to the vibrations of C–O and C=O from the carbonyl group. A new peak at 2.86 ppm in ^1^H NMR represented the methyl proton neighboring with C=O. The appearance of new peaks at 177.05 and 177.15 ppm in ^13^C NMR proves the substitution of the succinyl group in the κ-carrageenan structure. The elemental analysis was carried out to calculate the degree of substitution with the highest value of 1.78 at 24 h of reaction. The XRD diffractogram of derivatives exhibited a higher degree of crystallinity compared to pristine κ-carrageenan at 23.8% and 9.2%, respectively. Modification of κ-carrageenan with a succinyl group improved its interaction with ions and the conductivity of the salt solution compared to its pristine form. This work has a high potential to be applied in various applications such as sensors, drug delivery, and polymer electrolytes.

## 1. Introduction

The pattern of current research focuses on biopolymer polysaccharides as sensors, polymer electrolytes, and biological applications through the enhancement of active sites such as cellulose, chitosan, and carrageenan [1,2,3,4,5,6,7]. Modification of active sites in a biopolymer polysaccharide structure is vital for the enhancement of the polysaccharide’s performance. It has become a crucial way for researchers to determine methods to increase the interaction of polysaccharides with desired compounds. The current pattern of examination focuses on modifying the active site of a biopolymer polysaccharide with a more electronegative group such as a carboxymethyl group (–CH_3_COOH), a sulfate group (SO_3_H), or a phosphate group (–O–PO_3_H_3_). Mobarak et al. [6] synthesized carboxymethyl carrageenan and claimed substitution of the carboxymethyl group enhanced its physiochemical properties. They reported that the ionic conductivity of modified carrageenan increased by three orders of magnitude as compared to pristine carrageenan [1,6].

Meanwhile, Luo et al. [2] reported that carboxymethyl chitosan increased the adsorption of heavy metal ions compared to pristine chitosan. In 2014 and 2017, Wang et al. [5,7] modified *Artemisia sphaerocephala* polysaccharide by substituting the active site with a sulfate group with a higher electronegative atom. This modification showed improvement in biological activities and exhibited more significant antitumor events compare to the pristine form. Modified polysaccharide hydrogel with a high electronegative group was advantageous in drug delivery applications. Huang et al. [8] modified the active site of chitosan to produce a polysaccharide-based hydrogel via self-crosslinking. This modification aids in increasing the cross-link density of biopolymer hydrogel that indirectly enhances biological properties such as self-healing, anti-enzymatic hydrolysis, and the ability to control drug release, especially in much exudation tissue [8,9]. According to the literature studies that summarized earlier, the substitution of active sites with more electronegative groups improves the performance of the compound. Table 1 summarizes a few examples of functional groups substituted into polysaccharides and their applications. 

Carrageenan is an example of a biopolymer polysaccharide. Carrageenan is a natural biopolymer sulfated polysaccharide that consists of an alternating and repeating unit of four linked 3,6-anhydrous-α-D-galactose (D-unit) and three linked β-D-galactose (G-unit) components, which can be extracted from red seaweeds [4,18]. There are three primary forms of carrageenan: lambda- (λ), iota- (ι), and kappa (κ)-carrageenan. Because of its renewability, abundance in nature, and profusion of electronegative groups, researchers have made carrageenan a preferred compound for study, including its properties and reactivity compared to other compounds. Abdullah and co-workers in 2018 [19] reported that carrageenan possesses excellent features as a sensing material for heavy metal detection in comparison to chitosan due to the presence of more oxygen atoms in the κ-carrageenan structure [1,19]. Nevertheless, performance of the material can be further improved by modification of its structure—by substituting hydroxyl with a succinyl group in the carrageenan structure. Since κ-carrageenan consists of a high number of hydroxyl groups compared to other forms of carrageenan, it can be further studied to increase the degree of substitution for better performance outcomes.

Succinyl group consists of two carbonyl moieties, where it is expected to provide more electronegative sites compared to the carboxymethyl group. In work reported by Xin et al. [20] and Zhu et al. [11], succinyl cellulose and succinyl chitosan were successfully synthesized, respectively. However, the preparation methods were lengthy and tedious. In a work reported by Xin et al. [20], succinyl cellulose was successfully synthesized with a different degree of substitution (DS), and the adsorption behavior towards cations was studied. The adsorption capacity of cations increased with the increase of DS, demonstrating better performance. This can be explained by the introduction of a high degree of substitution of the succinyl group hence increasing the capacity for the binding of metal ions with the electronegative atoms in the succinyl group compared with a low DS. This phenomenon was also reported in polymer electrolyte and drug delivery applications, where the conductivity and drug loading was highest when using a biopolymer with a high DS [21,22]. Therefore, this research focused on the development of succinyl κ-carrageenan with a high degree of substitution using a simple one-step reaction, hence, reducing the complexity of the procedure.

Herein, we report on the synthesis of succinyl κ-carrageenan using a simple one-step reaction process by modifying the active site of biopolymer κ-carrageenan. We also managed to obtain a high degree of substitution as we varied the effect of the reaction time to increase the performance of κ-carrageenan. We analyzed the reactivity of the modified κ-carrageenan using conductivity analysis, and the results were compared with pristine κ-carrageenan. 

## 2. Materials and Method

### 2.1. Materials

Commercial-grade κ-carrageenan was obtained from Tacara Sdn Bhd (TA150) (Mw: 12 kDa). Succinic anhydride, acetone, acetonitrile, and ammonium chloride were purchased from Sigma–Aldrich (St. Louis, MO, USA). All solvents were used without further purification. Distilled water was used throughout the experiment.

### 2.2. Preparation of Succinyl κ-Carrageenan

Succinyl κ-carrageenan was prepared following previous methods developed by Mobarak et al. [23] and Zhu et al. [11] with some modifications. In this work, acetonitrile was used as the solvent for compound precipitation instead of ethanol. One gram of κ-carrageenan was dissolved in 200 mL of distilled water in a conical flask and stirred until all powdered κ-carrageenan turned into a gel-like solution. Two grams of succinic anhydride was dissolved into 20 mL of acetone and added drop-wise to the flask for 30 min at room temperature. The reaction was carried out for 4 h, 12 h, and 24 h at 40 °C, separately. After the response was completed, the mixture was cooled to room temperature. To precipitate the compound, excess acetone was added into the mixture, followed by acetonitrile. Finally, the solid was filtered and washed with acetonitrile to remove the unreacted succinic anhydride. The solid sample was kept dry in a desiccator for 12 h.

**Scheme 1:**
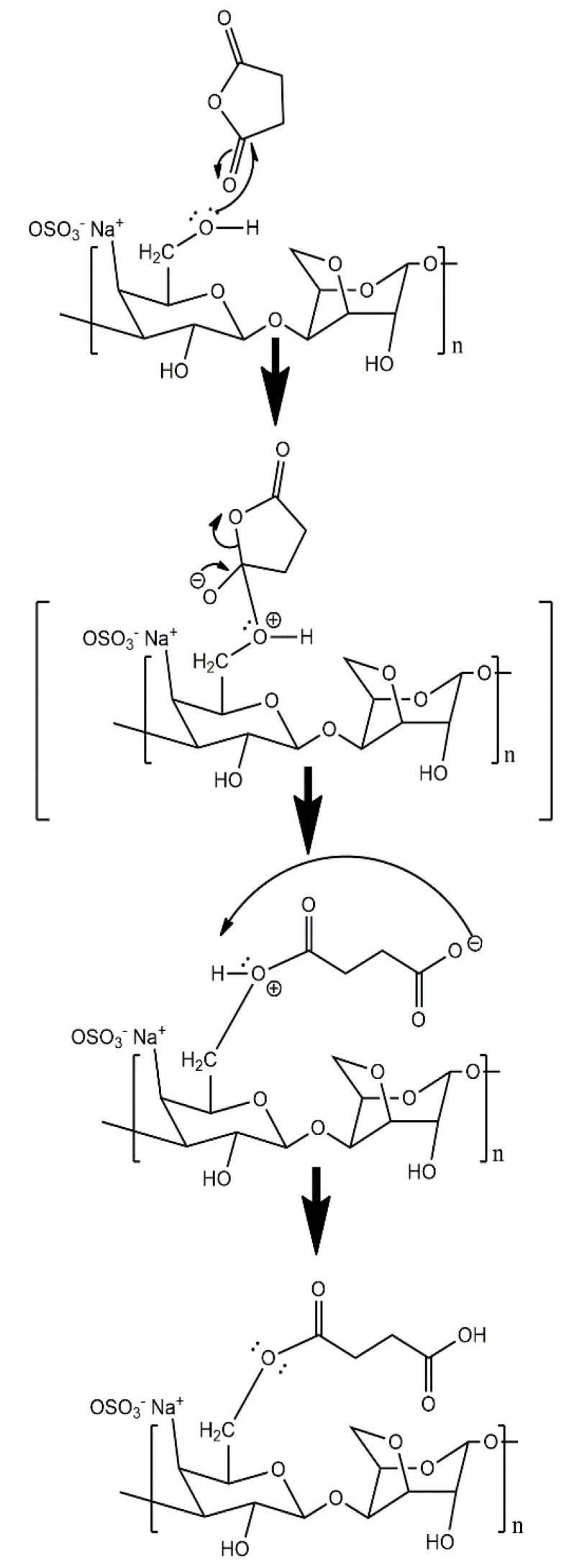
Proposed reaction mechanism scheme for the carboxylation of κ-carrageenan employed by the nucleophilic acyl substitution.

The overall proposed mechanism is shown in Scheme 1. Succinic anhydride acts as an electrophile that will provide a succinyl group into the κ-carrageenan skeleton. The addition of the hydroxyl group from κ-carrageenan into a succinic anhydride skeleton employed the nucleophilic acyl substitution process that follows the addition–elimination mechanism. The sp^2^ hybridized carbonyl carbon of succinic anhydride act as an electrophile, because it consists of an electronegative oxygen atom, resulting in the polarization of the carbon–oxygen double bond. Initially, the hydroxyl group of κ-carrageenan, which acts as a nucleophile, attacks the electrophile carbon of the carbonyl group in the succinic anhydrides producing a tetrahedral intermediate. Then, this is followed by expelling the leaving group through the ring-opening of the succinic anhydride skeleton to regenerate the carbonyl group. The reaction completed with the proton transfer [24]. 

## 3. Characterization

### 3.1. Fourier Transform Infra-Red Analysis (FTIR)

κ-Carrageenan and succinyl κ-carrageenan were characterized using a Cary 630 FTIR Agilent (Santa Clara, CA, USA) equipped with a diamond-attenuated total reflection accessory in the range of 4000 to 600 cm^−1^ with a scanning rate 1 cm^−1^. The data were recorded in transmittance mode. The characterization was carried out to observe and compare the change in intensity, chemical shift, and new peak formation in the spectrum of the modified κ-carrageenan and pristine κ-carrageenan. 

### 3.2. Nuclear Magnetic Resonance Analysis (NMR)

κ-Carrageenan and succinyl κ-carrageenan were further analyses using NMR to confirm the substitution of succinyl group into κ-carrageenan, the analysis of ^1^H and ^13^C NMR were carried out by using Bruker Avance 111 HD 600 MHz (Bruker, Rheinstetten, Germany) at 57 °C. Prior to NMR analysis, κ-carrageenan and succinyl κ-carrageenan were prepared by dissolving 5 mg of the sample into 600 μL D_2_O solvents followed by a few drops of deuterated acetic acid. The sample was sonicated and heated at 40 °C to facilitate the dissolution before analyzed with NMR.

### 3.3. X-Ray Diffraction Analysis (XRD) and Elemental Analysis

The crystallinity properties of succinyl κ-carrageenan and κ-carrageenan were studied using an X-ray diffractometer (Bruker D8 Quest SC-XRD, Billerica, MA, USA). The diffraction angle of 2θ was used, ranging from 5° to 80° with K_α2_ filtering. The XRD scan was carried out at a scan rate of 0.05° s^−1^. The crystallinity indexes were calculated using the software DIFFRAC EVA that was provided with the machine. The degree of substitution of succinyl κ-carrageenan was determined using the TruSpec Micro model CHNS elemental analysis (LECO, St. Joseph, MI, USA). The degree of substitution was calculated based on the following formula [23]:

(1) $$\mathrm{Degree}\;\mathrm{of}\;\mathrm{Substisution}=\frac{\left(\mathrm{Ratio}\;\mathrm{carbon}\;:{\mathrm{Sulphur}\;}_{\mathrm{Succinic}-k-\mathrm{carrageenan}}-\;\mathrm{Ratio}\;\mathrm{carbon}\;:{\mathrm{Sulphur}}_{k-\mathrm{carrageenan}}\right)}{\mathrm{Number}\;\mathrm{of}\;\mathrm{new}\;\mathrm{added}\;\mathrm{carbon}}$$

### 3.4. Conductivity Analysis

The conductivity (σ) measurement was carried out using a WTW 1F10-211911 Inolab Level 1 conductivity meter (measuring range 0.00–500 mS cm^−1^). This probe is composed of a built-in temperature sensor and has an automatic temperature compensation feature. The conductivity was measured based on the surrounding temperature during the measurement. The cell constant of InoLab was estimated by daily automatic calibration. For the calibration, the primary standard of an aqueous solution of potassium chloride (KCl) at a 0.01 mol dm^−3^ concentration with σ at 1413 µS cm^−1^ was used.

In this experiment, ammonium chloride (NH_4_Cl) was used as an electrolyte salt. A 1000 ppm stock solution of NH_4_Cl was prepared by diluting 1.486 g of dried NH_4_Cl salt in 500 mL of deionized water. Then a series of dilution was prepared from a stock solution ranging from 0.05 ppm to 10 ppm. A 0.05 g of the prepared κ-carrageenan and succinyl κ-carrageenan were immersed in 20 mL of prepared ammonium chloride solution concentration. Then, the mixtures were stirred for 5 h at room temperature. The mixtures were then filtered using a filter funnel, and the filtrate was analyzed using a conductivity meter at room temperature. The procedures were repeated three times to obtain the average conductivity. The measured conductivity of the NH_4_Cl solution immersed with κ-carrageenan and succinyl κ-carrageenan was compared with standard ammonium chloride solution.

In this experiment, the calculation of the infinite molar conductivity (Λ*_o_*) and the degree of dissociation of salt (α) was calculated based on the power-law method. The power-law method was used because the molar conductivity of the electrolyte became almost constant below a certain level of concentration (approximately ≈ 10^−6^) and due to the weak electrolyte system as well. The power-law method is composed of a double-logarithmic plot of Λ (molar conductivity) versus the *mol* in Equation (2) [25]:

(2) $$Log\;\Lambda =\;K^{\prime }+\;\gamma Log\;mol$$

(3) $$\alpha =\frac{\sigma }{\Lambda_{o}}$$

where Λ_o_ is the infinite molar conductivity, α is the degree of dissociation, and σ is conductivity at respective concentration. *K′* is the intercept, and *γ* is the slop of the double-logarithmic plot.

## 4. Results and Discussion

**Figure 1:**
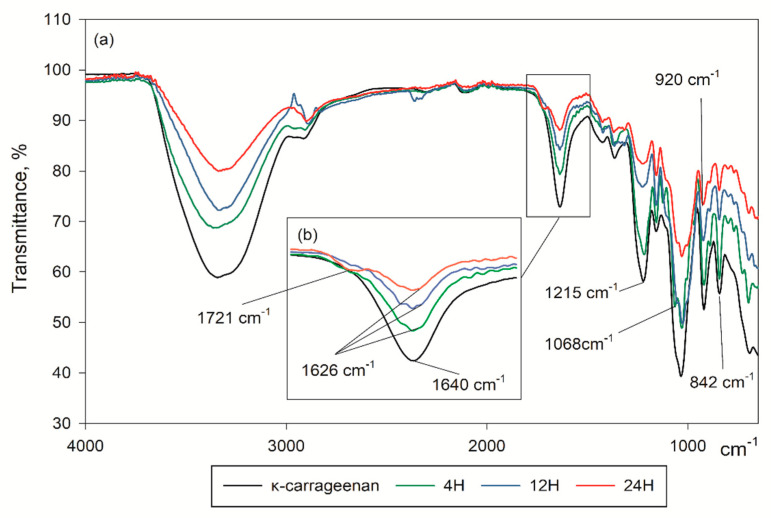
FTIR spectra for κ-carrageenan and succinyl κ-carrageenan with different reaction times. It shows a few new peaks that correspond to the mode of vibration in the succinyl group.

Figure 1 shows the FTIR spectra of κ-carrageenan and succinyl κ-carrageenan at various reaction times. The κ-carrageenan spectrum shows the typical peaks for κ-carrageenan at 1215 cm^−1^ and 842 cm^−1^ that represent the O=S=O symmetric vibration and C_4_–O–S stretching vibration, respectively [19,26]. Meanwhile, the appearances of C–O, C–H, and broad O–H stretches in κ-carrageenan were observed around 1040 cm^−1^, 2916 cm^−1^, and 3349 cm^−1^, respectively. The peak observed at 920 cm^−1^ represents C–O–C in 3,6-anhydrous-D-galactose. These peaks were very similar to the findings reported in the literature [4,26,27]. Meanwhile, the appearance of a new peak was observed at 1068 cm^−1^ for succinyl κ-carrageenan, representing the vibration of the C–O bond in the ester of the succinyl group [28]. 

**Figure 2:**
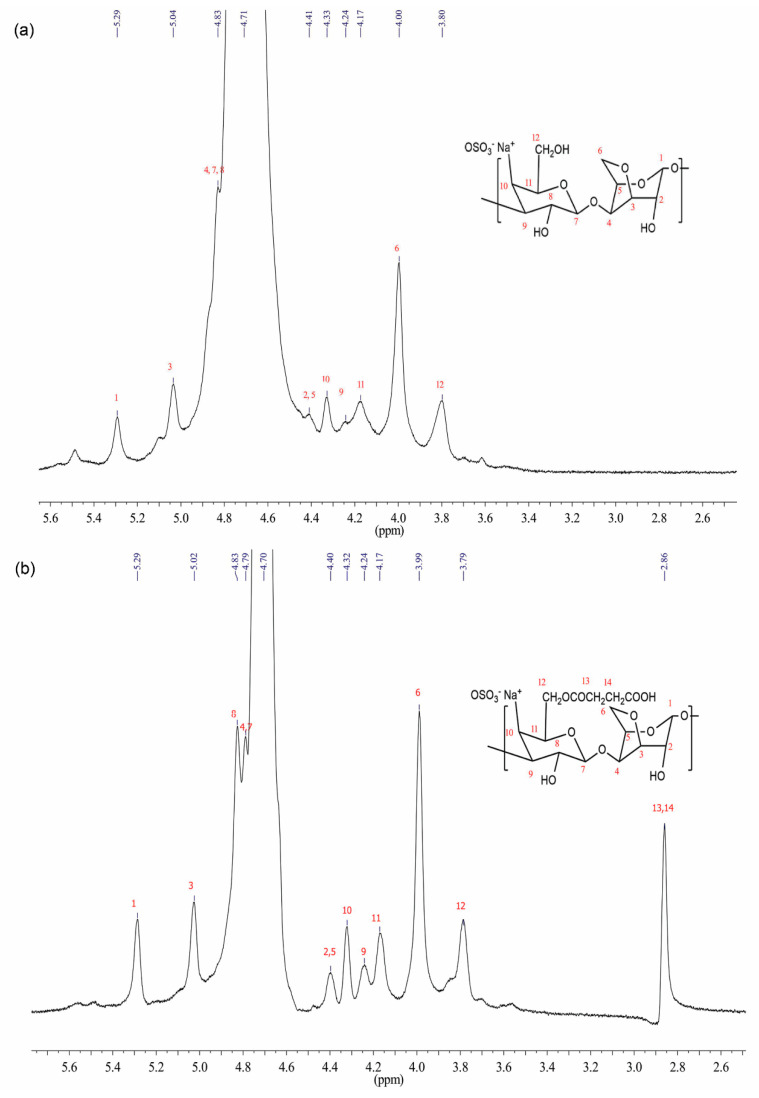
(**a**) ^1^H NMR spectrum for κ-carrageenan. (**b**) ^1^H NMR spectrum 24 h reaction of succinyl κ-carrageenan. The new peak spiked at 2.86 ppm, indicating the existence of a methylene group neighboring with the carbonyl group of ester and carboxylic acid from the succinyl group, proving the substitution of succinyl into the κ-carrageenan skeleton.

In addition, a new peak formation was also observed at 1626 cm^−1^ and overlapping with the structural water deformation peak at 1640 cm^−1^ [26]. This peak represents the asymmetrical stretching vibration mode of carboxylate anion (–COO–), which confirms the substitution of hydroxyl with the succinyl group in the κ-carrageenan structure. The peak at 1721 cm^−1^. represents the C=O vibration mode in ester and carboxylic acid, was observed for the 24 h reaction time. However, this peak was not spiked at 4 and 12 h reactions, indicating that the substitution of the succinyl group into κ-carrageenan was not complete. It was observed that the longer the reaction time, the higher the intensity of the peak. In general, the FTIR spectra (Figure 2) of succinyl κ-carrageenan showed the successful synthesis of succinyl κ-carrageenan. 

Different intensities of several peaks observed in FTIR spectra (Figure 1) revealed the variation in the degree of substitution (DS) with the reaction times. Table 2 shows the elemental percentage, the carbon-to-sulfur ratio and the DS in the κ-carrageenan structure at 4, 12, and 24 h of reaction calculated using the formula in Equation (1). It is predicted that the desulfation process does not occur in this synthesis because there is no alkali treatment applied [26]. The carbon-to-sulfur ratio in succinyl κ-carrageenan was higher compared to κ-carrageenan. This proves that succinic anhydride successfully reacted with κ-carrageenan hence increasing the percentage of carbon. The high percentage of carbon in succinyl κ-carrageenan shows that a high number of succinyl groups was substituted into the pristine κ-carrageenan structure. To further prove the substitution of succinic anhydride into κ-carrageenan, the DS was calculated from elemental analysis. It was observed that the DS value increased with the reaction time, where the highest DS of succinyl κ-carrageenan was 1.78 at 24 h of reaction.

To further confirm the substitution of the succinyl group into κ-carrageenan, the optimized degree of substitution of the synthesized compound was analyzed using ^1^H NMR and ^13^C NMR. The comparison between the ^1^H NMR spectrum of κ-carrageenan and the 24 h reaction of succinyl κ-carrageenan is shown in Figure 2a,b. Figure 2a shows the ^1^H NMR spectra of κ-carrageenan (D_2_O:CD_3_COOD), δ = 5.29 (H1), δ = 5.04 (H3, H8), δ = 4.83 (H4,H7,H8), δ = 4.41 (H2,H5), δ = 4.33 (H10), δ = 4.24 (H9), δ = 4.17 (H11), δ = 4.00 (H6), and δ = 3.80 (H12) are in agreement with the published reports [17,21]. Figure 2,b shows the ^1^H NMR spectra of the 24 h reaction for succinyl κ-carrageenan (D_2_O:CD_3_COOD), δ = 5.29 (H1), δ = 5.02 (H3), δ = 4.83 (H8), δ = 4.79 (H4, H7), δ = 4.40 (H2, H5), δ = 4.32 (H10), δ = 4.24 (H9), δ = 4.17 (H11), δ = 3.99 (H6), δ = 3.79 (H12), and δ = 2.86 (H13, H14). Succinyl κ-carrageenan shows a new peak at 2.86 ppm that corresponded to the proton hydrogen in methylene neighboring with the carbonyl group of ester and carboxylic acid from the succinyl group [28]. This proves that the hydroxyl group in κ-carrageenan was successfully substituted by the succinyl group.

**Figure 3:**
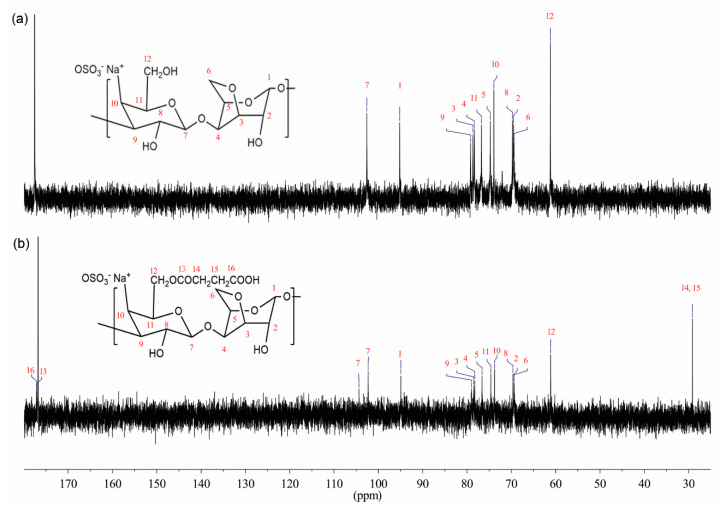
(**a**) ^13^C NMR spectrum for κ-carrageenan. (**b**) ^13^C NMR spectrum for the 24 h reaction of succinyl κ-carrageenan. The new peak spiked at 177.15, 177.05, and 29.15 ppm belong to the carbon in the succinyl group proving the successful substitution of the succinyl group in the κ-carrageenan structure.

Figure 3a shows the ^13^C NMR spectra for twelve carbons of κ-carrageenan, where ^13^C NMR (D_2_O:CD_3_COOD), δ = 102.43 (C7), δ = 95.04 (C1), δ = 79.13 (C9), δ = 78.63 (C3), δ = 78.31 (C4), δ = 76.69 (C5), δ = 74.70 (C11), δ = 73.91 (C10), δ = 69.75 (C8), δ = 69.50 (C2), δ = 69.37 (C6), and δ = 61.23 (C12). These peaks agree with the reported value in Kolender and Matulewicz [29] and Tranquilan-Aranilla et al. [27]. Figure 3b shows the intensity signal of the 24 h reaction of succinyl κ-carrageenan after the addition of four carbon atoms in the structure. The ^13^C NMR spectra of the 24 h reaction of succinyl κ-carrageenan in Figure 3b: ^13^C NMR (D_2_O:CD_3_COOD), δ = 177.15 (C16), 177.05 (C13), δ = 104.36, 102.32 (C7), δ = 95.92 (C1), δ = 79.02 (C9), δ = 78.49 (C3), δ = 78.24 (C4), δ = 76.61 (C5), δ = 74.62 (C11), δ = 73.79 (C10), δ = 69.63 (C8), δ = 69.41 (C2), δ = 69.37 (C6), δ = 61.14 (C12), and δ = 29.15 (C14, C15). 

The appearance of a new signal in succinyl κ-carrageenan at 177.15, 177.05, and 29.15 ppm confirmed the results obtained from the FTIR and ^1^H NMR analyses. The peak spiked at 177.15, and 177.05 ppm were assigned to the carboxylate anion (–CH_2_*COO–) of the carboxylic acid group and (–CH_2_*COO–) carbonyl carbon of ester group from the succinyl skeleton. Carbon atoms in these carbonyl groups are sp^2^ hybridized and attached to the electronegative oxygen, causing its deshielding and spiking at higher chemical shifts compared to the other carbons. Meanwhile, the peak spiked at 29.15 ppm, corresponding to the methylene carbon from the succinyl group (–*CH_2_COO–). These new peaks prove the substitution of the succinyl group into κ-carrageenan and supported by a previous report [26,27]. Most of the carbon in G4S and DA were slightly shielded after the substitution process, indicating the possible functionalization of the hydroxyl group at C2, C8, and C12 [26]. 

**Figure 4:**
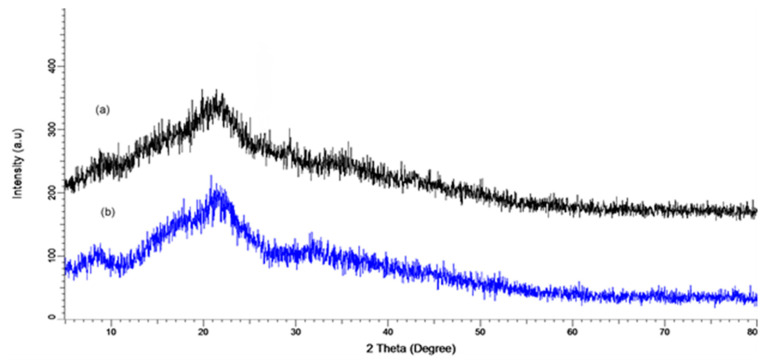
XRD diffractogram for (**a**) pristine κ-carrageenan and (**b**) succinyl κ-carrageenan at 24 h. The diffractogram shows both compounds are semi-crystal in nature.

Figure 4 shows the X-ray diffraction (XRD) analysis of pristine κ-carrageenan and succinyl κ-carrageenan at 24 h reaction. The broad hump in the range of 10°–25° in XRD diffractogram of pristine κ-carrageenan and succinyl κ-carrageenan reveals it is semi-crystalline [4,6]. However, the new diffraction peak formed at 2θ = 8° and 18° in succinyl κ-carrageenan diffractogram, showing that the addition of the succinyl group into κ-carrageenan slightly increased its crystallinity properties. The crystallinity index for succinyl κ-carrageenan was 23.8% compared to κ-carrageenan which was only 9.2%. This is due to the substitution of the succinyl group into κ-carrageenan, leading to the reorientation of the intermolecular and/or intramolecular structure resulting in a more ordered area. This trend is similar to the work reported by Wei et al. [4], where the addition of a new methylene phosphonic group in κ-carrageenan slightly increased the degree of crystallinity of the compound where this group also changed the inter and/or intramolecular structure of κ-carrageenan to form a more ordered region. A few studies have showed that the insertion of a charged group into a polysaccharide backbone will interrupt the intermolecular and/or intramolecular interaction. A simulation was performed in 2013 by Forget et al. [30] to study the effect of carboxylated κ-carrageenan. It showed the introduction of the carboxyl group switched in the secondary structure from an α-helical to β-sheet. The formation of extended β-sheet structure will form a more ordered region compared to coiled α-helical structure, proving the increase in the crystallinity percentage of succinyl κ-carrageenan compared to pristine succinyl κ-carrageenan. 

**Figure 5:**
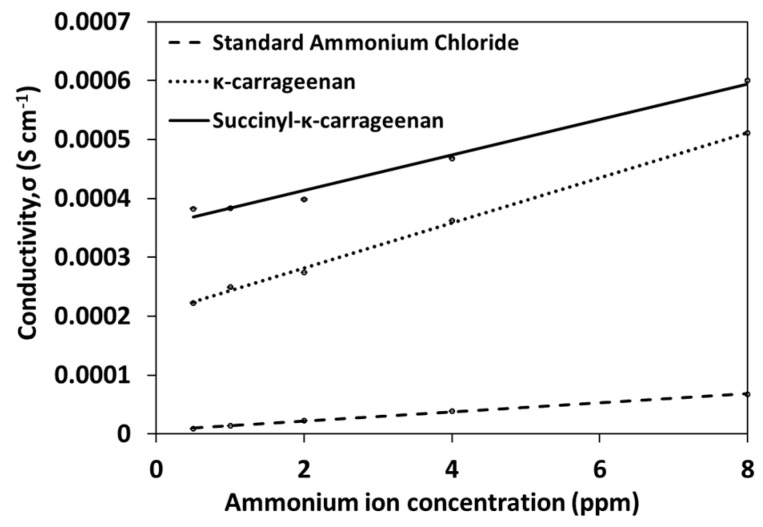
Ionic conductivity of standard NH_4_Cl solution, κ-carrageenan–NH_4_Cl solution, and succinyl κ-carrageenan–NH_4_Cl solution at room temperature.

The performance of modified κ-carrageenan was observed based on the conductivity behavior of salt upon reaction with succinyl κ-carrageenan. The ionic conductivity of standard NH_4_Cl, pristine, and succinyl κ-carrageenan immersed in NH_4_Cl are illustrated in Figure 5. The conductivity of all salt solutions raised with the increase of ammonium concentration. However, the ionic conductivity of the succinyl κ-carrageenan–NH_4_Cl solution was higher compared to the κ-carrageenan–NH_4_Cl solution and standard NH_4_Cl solution. This was due to the increase in the infinite molar conductivity and dissociation degree of NH_4_Cl solution after reaction with succinyl κ-carrageenan.

**Figure 6:**
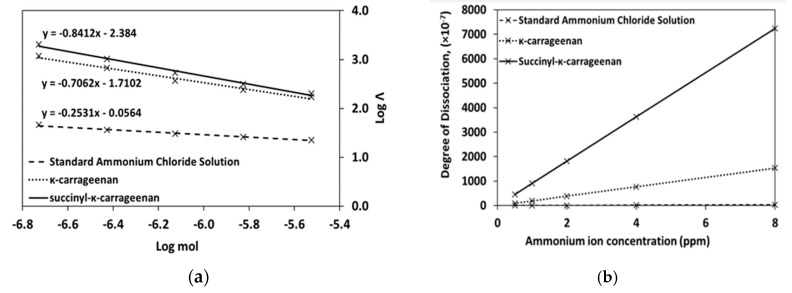
(**a**) Double logarithmic plot of log Λ versus log (mol) of NH_4_Cl in standard NH_4_Cl, κ-carrageenan–NH_4_Cl and succinyl κ-carrageenan–NH_4_Cl solutions at room temperature; (**b**) degree of dissociation (α) of standard NH_4_Cl, κ-carrageenan–NH_4_Cl, and succinyl κ-carrageenan–NH_4_Cl solutions at room temperature.

The hypothesis of the infinite molar conductivity and dissociation degree of NH_4_Cl was further proved through a mathematical calculation using the formula in Equations (2) and (3). The output of the calculation is depicted in Figure 6a,b. Based on the results, succinyl κ-carrageenan has a higher degree of dissociation compared to pristine κ-carrageenan and standard NH_4_Cl. The presence of more electronegative atoms in succinyl κ-carrageenan compared to pristine induced the dissociation of salt through the formation of ion-dipole or coordination of the interaction among oxygen atoms with NH_4_^+^ ion in the salt solution [31,32]. Meanwhile, the formation of strong hydrogen bonding between –OH groups in succinyl κ-carrageenan with Cl− ions of NH_4_Cl, leads to the dissociation of NH_4_Cl salt. This phenomenon caused the separation of salt into ion forms, hence, increasing the ion concentration in the solution. Lu et al. [33] confirmed the presence of hydrogen bond interaction between –OH in cellulose with anion in salt solution. This phenomenon caused the increment of NH_4_Cl solution conductivity compared to the standard NH_4_Cl solution. A similar trend and findings were also reported in previous works [31,33]. 

## 5. Conclusions

Succinyl κ-carrageenan was successfully synthesized by reacting κ-carrageenan and succinic anhydride using a one-step modification reaction. Characterization of succinyl κ-carrageenan using FTIR analysis confirmed the substitution of the succinic group into a κ-carrageenan backbone. The ^1^H and ^13^C NMR analysis revealed the functionalization of the hydroxyl group of κ-carrageenan with a succinyl group. The XRD analysis showed that the succinyl κ-carrageenan was semi-crystalline with a higher degree of crystallinity compared to κ-carrageenan. The degree of substitution was calculated using elemental analysis with the highest value of 1.78 at 24 h of reaction. Modification of κ-carrageenan increased the number of electronegative contents in the biopolymer backbone compared to the pristine κ-carrageenan. In parallel, it enhanced the performance of the material toward analytes. This phenomenon was confirmed when succinyl κ-carrageenan showed greater interaction with the NH_4_Cl solution compared to the pristine solution through conductivity analysis. The interaction between electronegative and hydroxyl groups in succinyl κ-carrageenan with ion in the NH_4_Cl solution increased the conductivity behavior of the NH_4_Cl solution. This is due to the increment of the dissociation degree of salt compared to the pristine κ-carrageenan and standard NH_4_Cl solutions. The introduction of the succinyl group into κ-carrageenan will provide advantages in various applications. It is expected that the synthesized derivative compound has high potential for future research and in various applications such as sensors, drug delivery, and polymer electrolytes. This compound can be further characterized using metal sorption isotherm study to investigate its ability as metal sorption, electrical impedance to study its ability to conduct electricity, and drug-releasing assay (release kinetic) to study its ability to retain/deliver the drug. 

## Figures and Tables

**Table 1:** Application of modified polysaccharide.

Functional Groups	Biopolymer Polysaccharides	Applications	References
Carboxymethyl	Chitosan, chitin, cellulose, κ-carrageenan	Polymer electrolyte, ion removal, drug delivery	[1,2,3,9,10,11]
Phosphate	κ-carrageenan, chitosan, cellulose	Polymer electrolyte, medical, ion removal, drug removal	[4,12,13,14]
Sulfate	*Artemisia sphaerocephala* polysaccharide, chitosan, cellulose, alginate	Biological and medical application, ion removal	[5,15,16,17]

**Table 2:** Degree of substitution of κ-carrageenan and succinyl κ-carrageenan at various reaction times.

Sample	Carbon, (%)	Sulfur, (%)	Oxygen, (%)	Ratio, (C/S)	DS
κ-Carrageenan	27.54	61.6	5.52	4.99	-
Succinyl κ-carrageenan (4 h)	26.94	62.16	5.17	5.21	0.06
Succinyl κ-carrageenan (12 h)	32.50	57.86	3.10	10.48	1.37
Succinyl κ-carrageenan (24 h)	33.17	57.88	2.74	12.10	1.78
